# Development of a Murine Model of Early Sepsis in Diet-Induced Obesity

**DOI:** 10.1155/2014/719853

**Published:** 2014-05-25

**Authors:** Momina Khan, Amanda L. Patrick, Alison E. Fox-Robichaud, The Canadian Critical Care Translational Biology Group

**Affiliations:** ^1^Department of Medicine, Thrombosis and Atherosclerosis Research Institute, McMaster University, DBRI C5-106, 237 Barton Street East, Hamilton, ON, Canada L8L 2X2; ^2^The Critical Care Translational Biology Group, 555 University Avenue RM 2830A, Toronto, ON, Canada M5G 1X8

## Abstract

Sepsis, a global health issue, is the most common cause of mortality in the intensive care unit. The aim of this study was to develop a new model of sepsis that investigates the impact of prolonged western diet (WD) induced obesity on the response to early sepsis. Male C57BL/6 mice were fed either a high fat WD or normal chow diet (NCD) for 6, 15, or 27 weeks. Septic obese mice at 15 and 27 weeks had significantly lower levels of lung myeloperoxidase (26.3 ± 3.80 U/mg tissue) compared to age matched ad lib (44.1 ± 2.86 U/mg tissue) and diet restricted (63.2 ± 5.60 U/mg tissue) controls. Low levels of lung inflammation were not associated with changes in hepatic cytokines and oxidative stress levels. Obese mice had significantly (*P* < 0.0001) larger livers compared to controls. Histological examination of the livers demonstrated that WD fed mice had increased inflammation with pronounced fat infiltration, steatosis, and hepatocyte ballooning. Using this model of prolonged exposure to high fat diet we have data that agree with recent clinical observations suggesting obese individuals are protected from sepsis-induced lung injury. This model will allow us to investigate the links between damage to the hepatic microcirculation, immune response, and lung injury.

## 1. Introduction

Given the current obesity epidemic, the ratio of overweight and obese patients with critical illness is increasing rapidly; however, how obesity shapes critical illness and immune response to infection is understudied. Prolonged nutrient surplus causes macrophages to accumulate in the liver and intramuscular visceral fat, triggering synthesis of proinflammatory mediators such as interleukin-6 (IL-6), tumor necrosis factor alpha (TNF-*α*), macrophage chemoattractant protein-1 (MCP-1), matrix metalloproteinases (MMPs), and fatty acid binding protein [1] that contribute to the development of cardiovascular diseases and metabolic syndrome. Cytokine and hormone production in adipocytes, as well as the release of nitric oxide and reactive nitrogen species all play a role in disruption of normal insulin signalling contributing to hyperglycemia and insulin resistance [2]. It is essential to characterize the extent of hyperglycemia as well as the state of insulin resistance in order to understand the implications of obesity versus type II diabetes.

The link between metabolic disorders and inflammation was first discovered in 1993 by Hotamisligil et al., where they reported adipocytes to continuously express TNF-*α* and that enhanced TNF-*α* levels in obese mice could be reversed with weight loss [3]. From clinical studies it is known that morbidly obese patients have reduced adiponectin levels and elevated plasminogen activator inhibitor 1 (PAI-1), interleukin-1*α* (IL-1*α*), IL-6, and TNF-*α* levels [4–7].

Since obesity alone is considered a proinflammatory state, exposure to an external inflammatory stimulus, such as an infectious agent, is assumed to further exacerbate the inflammatory response with worse outcomes for obese compared to normal weight (BMI < 25 Kg/m^2^) patients. Sepsis is a critical illness characterized by systemic inflammatory response of host immune system to infection. Progression to septic shock and the development of multiorgan dysfunction are associated with mortality rates as high as 60% [8].

Several studies have investigated implications of obesity for outcomes of critical illness. However, the majority of the studies utilize nonclinically relevant animal models that contribute to lack of consistency between findings. Transgenic obese mice with a leptin deficiency (ob/ob) are commonly used to study obesity [9–11]. These mice do not mimic human obesity since the genetic causes of the condition are very rare and in addition, leptin deficiency is independently associated with defects in the immune function [12, 13]. Models of diet induced obesity (DIO) have been utilized to investigate immune responses to bacterial infections; however, the majority examine short-term effects [14]. An exception is a study by Lawrence et al., where mice were fed a high fat diet for 20 weeks and sepsis was induced with lipopolysaccharide (LPS) [15]. The main limitation of using LPS to induce sepsis is that it initiates very different responses in mice compared to patients in the clinical settings and therefore lacks clinical relevance [16]. To our knowledge there has been no description of organ specific inflammatory response in prolonged DIO in a clinically relevant murine model of sepsis. Therefore, this study will describe effects of early sepsis on hepatic and pulmonary inflammation, oxidative stress, and subsequent organ dysfunction in a clinically relevant murine model of prolonged diet-induced obesity.

## 2. Methods

### 2.1. Experimental Animals

C57BL/6 mice (3–5 weeks old) were ordered from Taconic (Germantown, NY). Animals were housed in a clean room with regulated temperature and a 12-hour light-dark cycle. They were fed a high fat western diet (WD) (from Dyets Inc., “Modified choline deficient diet,” Bethlehem, PA) for 6, 15, or 27 weeks with free access to water prior the induction of sepsis. There were two sets of age matched controls; both were fed normal chow diet (NCD). One group had* ad libitum* access to food (NCD-AL), whereas the other group was 30% diet restricted (NCD-DR). Diet restriction was introduced gradually after approximately 2.5 months of age as previously done by Bonkowski et al., 2006 [17]. A group of control mice labelled “diet only” was incorporated in this study. These mice did not undergo sepsis or sham surgery. Approximately 5-6 mice were used for most experiments. Data was pooled if similar. All animal protocols were approved by the Animal Rights Ethics Board at McMaster University in accordance with the Canadian Council of Animal Care regulations.

### 2.2. Sepsis Model: Cecal Ligation and Puncture

Polymicrobial sepsis was induced using cecal ligation and puncture (CLP). Isoflurane anaesthetized mice were weighed and injected with analgesic and 2 mL Ringer's Lactate subcutaneously. A catheter was inserted into the right jugular vein, secured with 4-0 sutures and tunnelled to the back of the neck. The cecum was exposed through a one centimeter wide incision along the midline abdomen, a one centimetre distal to the ileal cecal junction ligated and punctured once with an 18 G needle. Mice received 500 *μ*L of Ringer's Lactate intravenously right after surgery as well as four hours later. Six hours postsurgery, anaesthetized mice were sacrificed using the cervical dislocation technique. Plasma as well as tissue samples were collected and snap frozen in liquid nitrogen. Sham surgery only involved catheterization of the right jugular vein followed by incision to the abdominal muscle; however, no ligation or puncturing of the cecum was involved.

### 2.3. White Blood Cell Count (WBC)

White blood cell count was utilized to quantify the number of leukocytes in blood samples obtained from all experimental groups. 25 *μ*L of blood was mixed with 30% acetic acid and crystal violet solution. Total white blood cells were counted using a hemocytometer and expressed as cells/L.

### 2.4. Lung Myeloperoxidase Activity (MPO) Assay

Lung samples were snap frozen in liquid nitrogen and stored at −80°C. For the assay, samples were homogenized for 30 seconds and centrifuged at 10,000 rpm for 10 minutes. The pellet was resuspended in HTAB, homogenized again for 30 seconds, and centrifuged at 30000 rpm for 15 minutes. 7 *μ*L of the sample was added in triplicate to a 96 well plate. 50 *μ*L of 0.021% H_2_O_2_ solution was added to a cocktail mix of distilled water, potassium phosphate buffer, and O-dianisidine and added to each well. Changes in absorbance were measured at 450 nm by a spectrophotometer for 90 seconds. Results are represented in units of MPO activity per gram of tissue.

### 2.5. Glucose Tolerance Test (GTT)

Mice were fasted for 6 hours and administered with an intraperitoneal injection of 2 g/kg of 20% glucose solution. Blood glucose levels were measured from the tail vein at basal as well as 15 minute intervals for the first hour followed by a final measurement at the 2-hour time point using a standard glucometer.

### 2.6. Insulin Tolerance Test (ITT)

Random-fed mice were administered with 0.75 U/kg of insulin intraperitoneally. Glucose levels were measured from the tail vein at basal as well as at 15, 30, 60, and 120 minute time points using a standard glucometer.

### 2.7. Glutathione Assay

Glutathione assay was performed using kits purchased from Cayman Chemical (distributed by Cedarlane Labs, Burlington, ON), according to the manufacturer's instructions.

### 2.8. Cytokine Assay

Hepatic cytokine and chemokine levels were measured with Bioplex assay kits (from Bio-Rad, Mississauga, ON) according to the manufacturer's instructions.

### 2.9. Statistical Analysis

Data are expressed as mean and SEM. All data was analyzed using ANOVA (with Bonferroni correction of 0.05) and Student's *t*-test. A *P* value greater than 0.05 was considered significant.

## 3. Results

### 3.1. WD Feeding Metabolic Response

**Figure 1:**
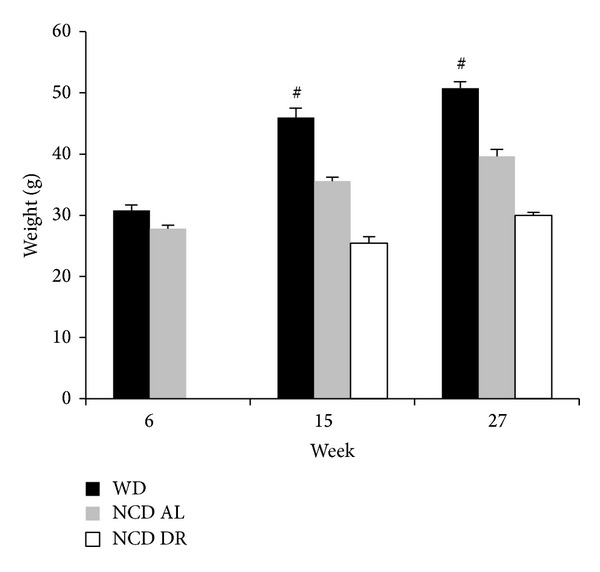
Average weight of each diet group at their respective end points. Data is presented as the mean (SE) (*n* = 5/group). ^#^ *P* < 0.0001 WD compared to NCD AL and NCD DR. ^∧^ *P* < 0.0001 NCD AL compared to NCD DR.

**Figure 2:**
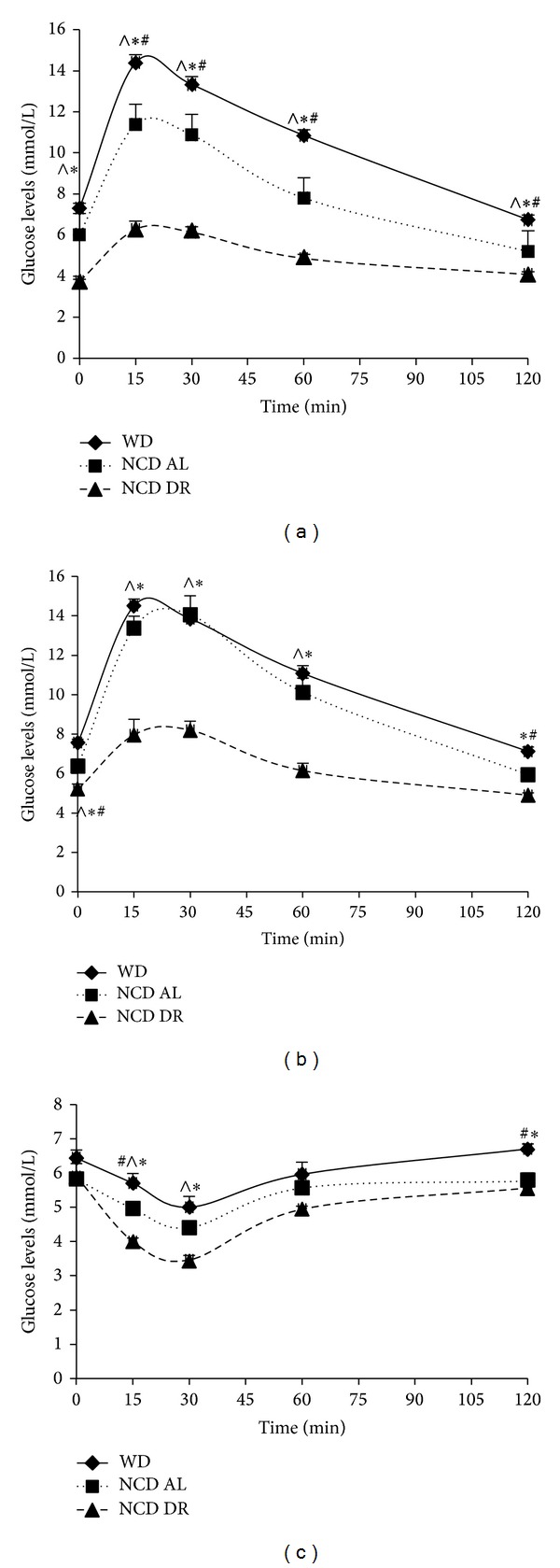
Glucose test at 15 weeks (a), glucose tolerance test at 27 weeks (b), and insulin tolerance test at 27 weeks (c). Results are expressed as changes in glucose levels after an I.P challenge of 2 g/kg glucose and 0.75 U/kg insulin. Data is presented as the mean (SE) (*n* = 5 for NCD AL and NCD DR and *n* = 15 for WD for glucose tolerance test and *n* = 5/group for insulin tolerance test). ^#^ *P* < 0.05 for WD compared to NCD AL, *P < 0.05 for WD compared to NCD DR, ^∧^ *P* < 0.05 NCD AL compared to NCD DR.

Mice fed WD developed obesity by gaining significantly more weight (50.8 g ± 1.05 at 27 weeks) compared to age matched controls (39.6 g ± 1.18) as shown in Figure 1. Due to the increase in weight, there were also changes in response to glucose and insulin challenges. WD fed mice at 15 weeks had significantly higher glucose levels after a glucose challenge compared to both control groups as seen in Figure 2(a), suggesting glucose intolerance. After 27 weeks, however, normal chow diet fed ad lib (NCD AL) control group also showed the same response to both glucose and insulin challenges as WD fed obese mice as seen in Figures 2(b) and 2(c). Normal chow diet restricted (NCD DR) control group, however, continuously had significantly lower glucose levels compared to the other two groups. Therefore, as expected increase in body mass was strongly associated with glucose intolerance and symptoms of metabolic syndrome not only in the WD fed obese mice but also in the ad lib controls after 27 weeks on WD. None of the mice developed overt diabetes.

### 3.2. Lung MPO Levels in Obese Septic Mice

**Figure 3:**
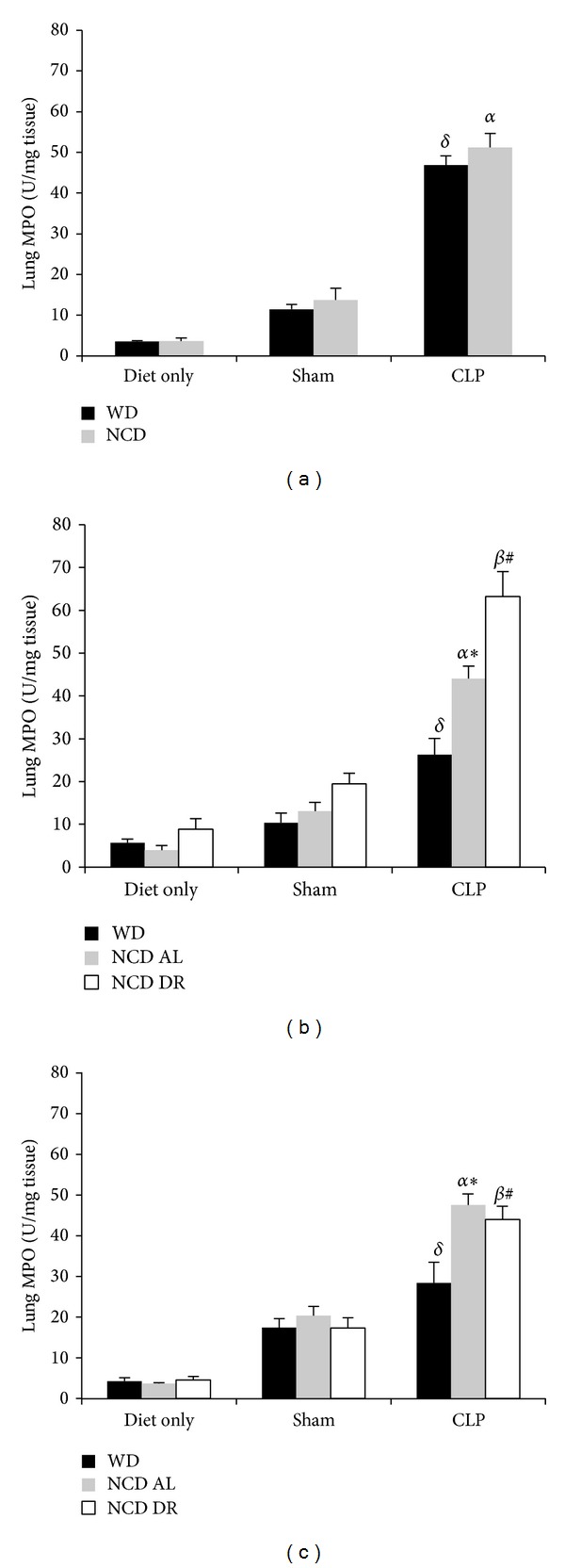
Lung MPO levels after 6 weeks (a), 15 weeks (b), and 27 weeks (c). Data is presented as the mean (SE) (*n* = 5/group). ^*δ*^ *P* < 0.05 for WD CLP compared to WD sham and diet only. ^*α*^ *P* < 0.001 for NCD AL CLP compared to NCD diet only and shams. ^*β*^ *P* < 0.0001 for NCD DR CLP compared to NCD DR sham and diet only. *P < 0.01 for WD CLP compared to NCD AL CLP. ^#^ *P* < 0.01 for WD CLP compared to NCD DR CLP.

Lung MPO levels were quantified for all groups as a measure of airway inflammation. As anticipated MPO levels were the lowest in the diet only groups and the highest during sepsis for all diet and age groups. Obese septic mice at 6 weeks did not have a significant difference in MPO levels (46.9 ± 2.20 U/mg tissue) compared to controls (51.2 ± 3.38 U/mg tissue) as seen in Figure 3(a). However, obese mice at 15 weeks had significantly lower lung MPO (26.3 ± 3.80 U/mg tissue) compared to NCD AL (44.1 ± 2.86 U/mg tissue, *P* < 0.01) and NCD DR controls (63.2 ± 5.60 U/mg tissue, *P* < 0.0001) as seen in Figure 3(b). Similar results were found at 27 weeks where obese septic mice again had significantly (*P* < 0.01) lower MPO levels (28.3 ± 5.08 U/mg tissue) compared to NCD AL (47.5 ± 2.70 U/mg tissue) and NCD DR (43.9 ± 3.29 U/mg tissue) as illustrated in Figure 3(c). Therefore, WD induced obesity is associated with low MPO levels and thus less airway inflammation during early sepsis.

### 3.3. Hepatic Oxidative Stress and Inflammation in Obese Septic Mice

**Figure 4:**
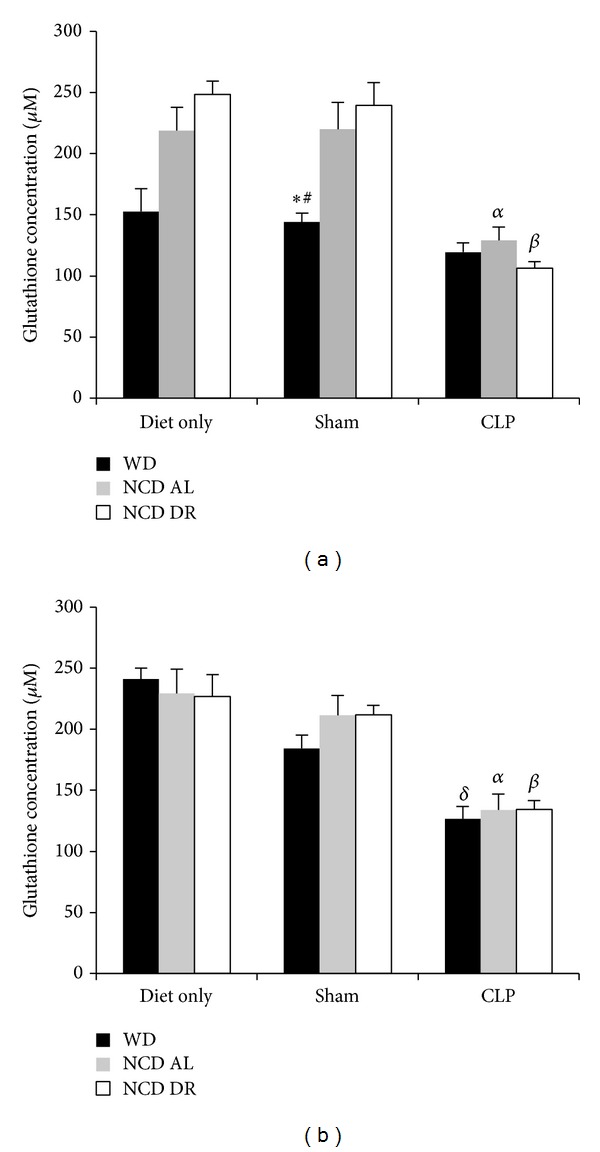
Hepatic glutathione levels after 15 weeks (a) and 27 weeks (b). Data is presented as the mean (SE) (*n* = 4 for naïve and shams and *n* = 5 for CLP groups). ^*δ*^ *P* < 0.0001 for WD CLP versus WD diet only. ^*α*^ *P* < 0.05 for NCD AL CLP compared to NCD AL sham and diet only. ^*β*^ *P* < 0.01 for NCD DR CLP compared to NCD DR sham and diet only. ^#^ *P* < 0.05 for NCD AL sham compared to WD sham. *P < 0.05 for NCD DR sham compared to WD sham.

Hepatic glutathione was quantified as a marker of oxidative stress. Obese mice at 15 weeks had no significant difference in terms of glutathione levels in the western diet only group (153 ± 18.5 *μ*M) and shams (144 ± 7.32 *μ*M) compared to septic (119 ± 7.66 *μ*M) mice as shown in Figure 4(a).  However, obese mice at 27 weeks had significantly lower (*P* < 0.0001) glutathione concentration in septic mice (127 ± 10.3 *μ*M) compared to western diet only (241 ± 8.93 *μ*M) and shams (184 ± 11.0 *μ*M) as shown in Figure 4(b). In terms of controls, in both NCD AL and NCD DR groups at both 15 and 27 weeks, septic mice (129 ± 11.3 *μ*M, 134 ± 12.3 *μ*M) had significantly (*P* < 0.05) lower glutathione levels compared to shams (220 ± 22.0 *μ*M, 212 ± 16.5 *μ*M) and respective diet only groups (219 ± 19.0 *μ*M, 230 ± 20.0 *μ*M) mice. There was no significant difference in terms of hepatic glutathione levels between obese septic mice compared to normal weight or diet restricted septic mice at any age.

**Figure 5:**
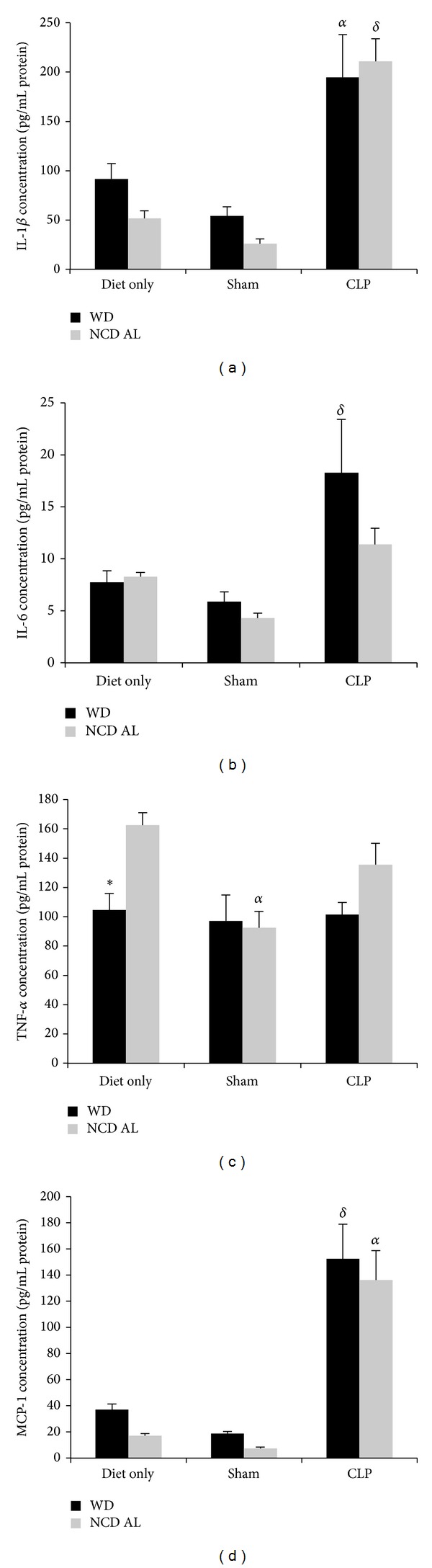
Hepatic chemokine and cytokine levels after 27 weeks: IL-1*β* (a), IL-6 (b), TNF-*α* (c), and MCP-1 (d). Data is presented as the mean (SE) (*n* = 6/group). ^*δ*^ *P* < 0.05 for WD CLP compared to WD sham and diet only. ^*α*^ *P* < 0.01 for NCD AL CLP compared to NCD AL diet only and shams. ^∗^ *P* < 0.05 for NCD AL diet only compared to WD diet only.

Oxidative stress and synthesis of glutathione is closely linked with cytokine expression. Therefore, hepatic interleukin-1 beta (IL-1*β*), IL-6, TNF-*α*, and MCP-1 levels were quantified in a different group of mice fed WD for 27 weeks, as illustrated in Figures 5(a)–5(d). IL-1*β*, IL-6, and MCP-1 levels were all upregulated in sepsis in both obese mice and NCD AL controls as seen in Figures 5(a), 5(b), and 5(d).  However, there were no significant differences in hepatic proinflammatory cytokine levels in obese compared to control septic mice. Hepatic keratinocyte chemoattractant (KC) expression was also measured (data not shown);  however, again there was no significant difference between septic obese mice and controls.

### 3.4. Hepatic Histology and Systemic Inflammation

**Figure 6:**
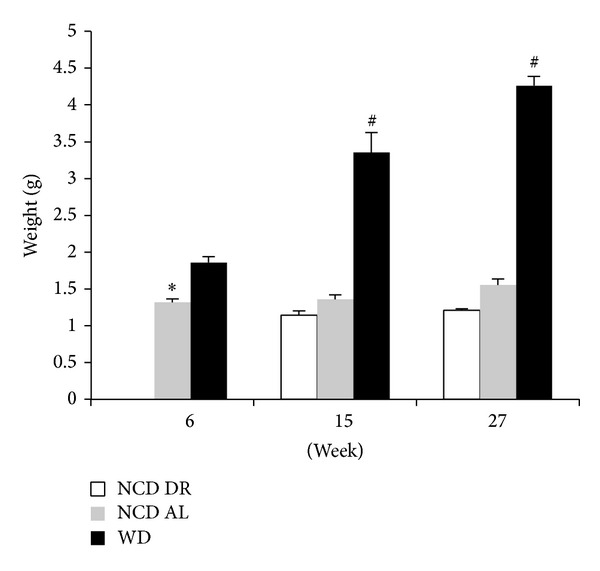
Liver weights of each diet group at their respective end points. Data is presented as the mean (SE) (*n* = 15/group). ^#^ *P* < 0.0001 for WD compared to NCD AL and NCD DR. *P < 0.01 for WD compared to NCD AL at 6 weeks.

**Figure 7:**
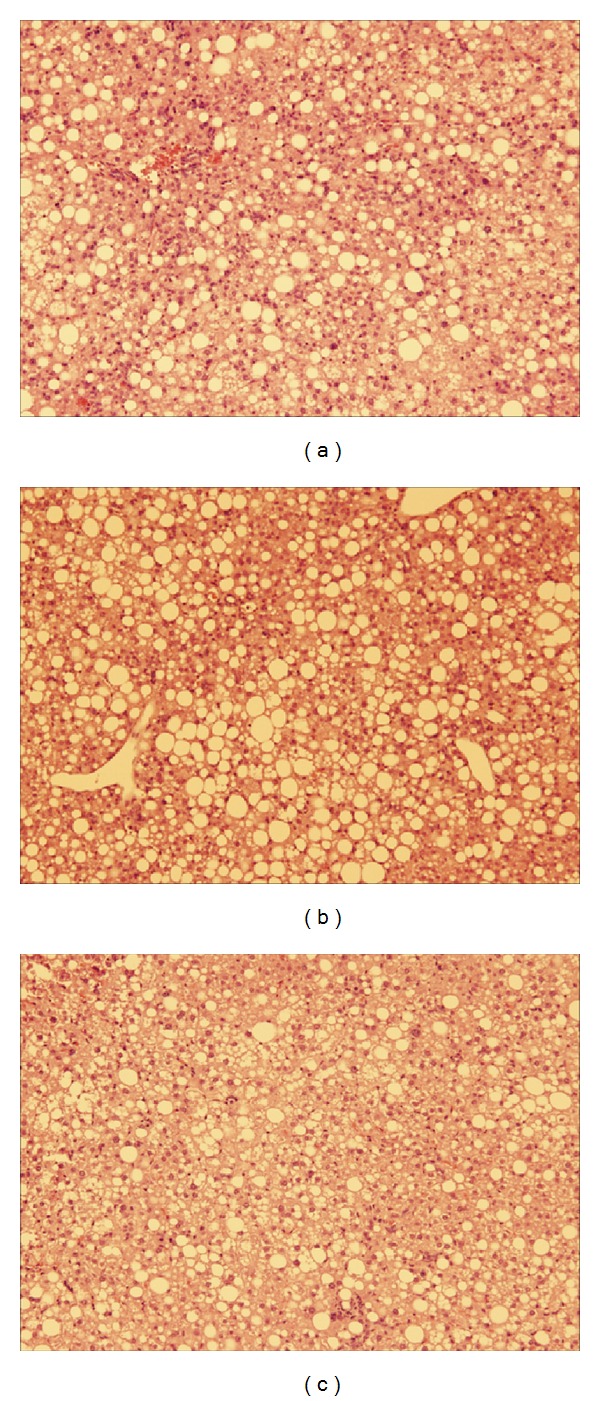
Hepatic histology of obese mice at 27 weeks: (a) induced with sepsis, (b) sham group, and (c) diet only group. H&E staining of the liver ×100 magnification.

**Figure 8:**
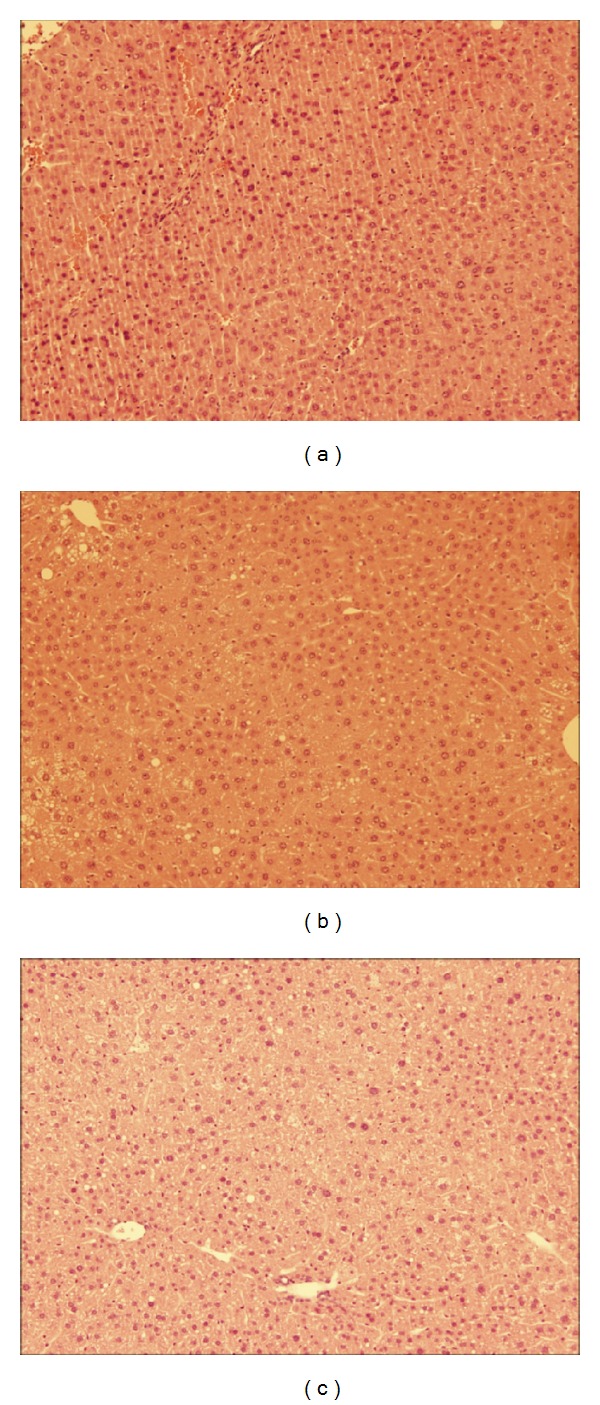
Hepatic histology of NCD AL control group at 27 weeks: (a) induced with sepsis, (b) sham group, and (c) diet only group. H&E staining of the liver ×100 magnification.

**Figure 9:**
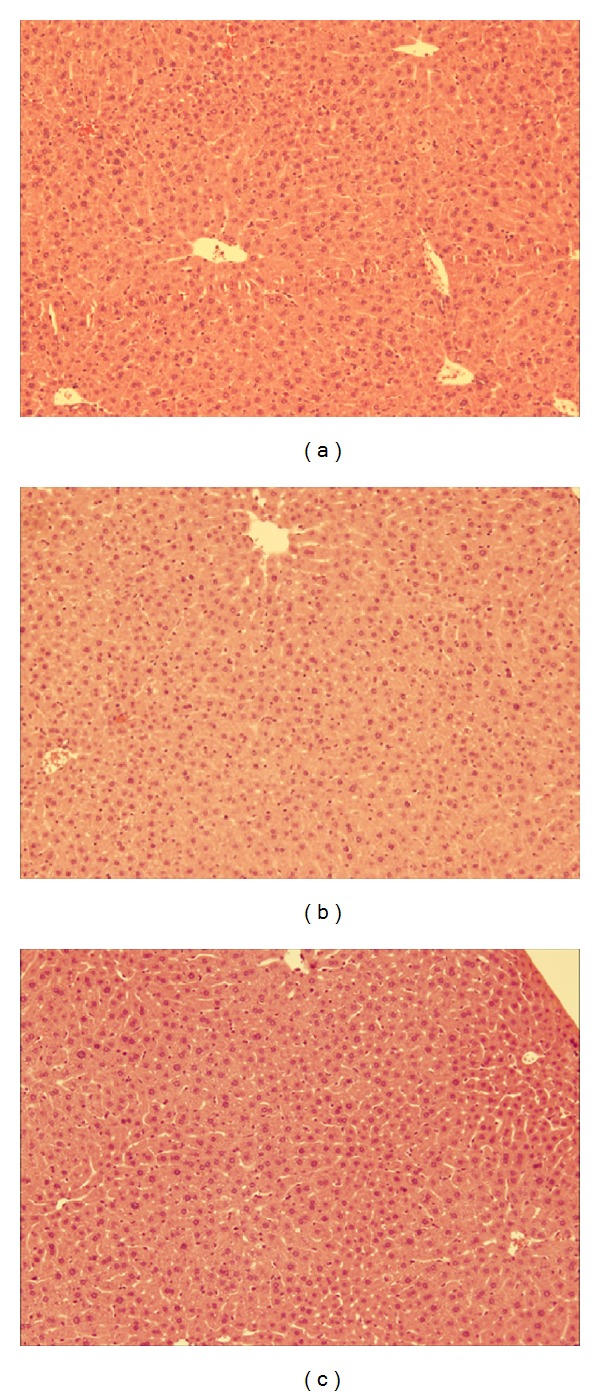
Hepatic histology of NCD DR control group at 27 weeks: (a) induced with sepsis, (b) sham group, and (c) diet only group. H&E staining of the liver ×100 magnification.

An increase in body mass was also reflected in age-dependent changes in hepatic structure and morphology. Mice fed WD had progressively larger livers the longer they were on the diet as shown in Figure 6, where obese mice at 27 weeks had significantly larger liver (4.23 ± 1.07 g) compared to obese mice at 6 (1.86 ± 0.0820 g) and 15 (3.40 ± 0.257 g) weeks. The controls also followed the same trend and, however, had significantly smaller livers compared to WD fed groups. Liver samples were stained with hematoxylin and eosin (H&E) to score damage to the hepatic microvasculature. As shown in Figures 7(a)–7(c), WD fed mice had greater damage to the liver with pronounced lipid deposition between and inside the hepatocytes as well as hepatocyte ballooning and neutrophil infiltration independent of surgery. Both groups of controls had very few or no lipid deposits in or around the hepatocytes as shown in Figures 8 and 9.

**Figure 10:**
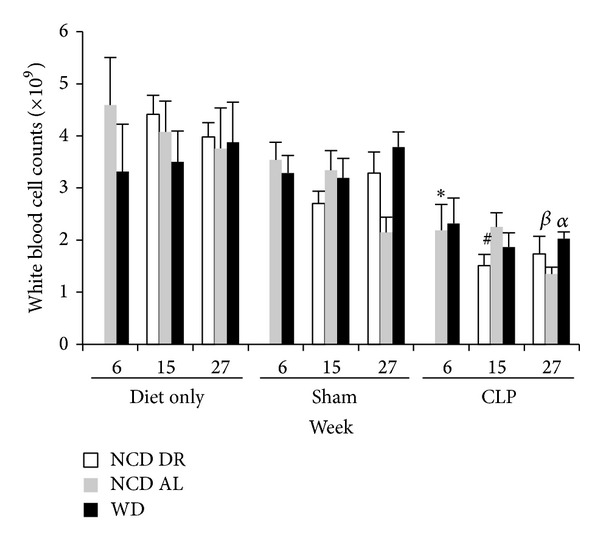
White blood cell counts for all diet groups at their respective end points. Data is presented as the mean (SE) (*n* = 5/group). *P < 0.05 for NCD AL diet only versus NCD AL CLP at 6 weeks. ^#^ *P* < 0.0001 for NCD DR diet only versus NCD DR CLP at 15 weeks. ^*α*^ *P* < 0.01 for NCD AL diet only versus NCD AL CLP at 27 weeks. ^*β*^ *P* < 0.05 for NCD DR diet only versus NCD DR CLP at 27 weeks.

As shown in Figure 10, the diet only groups of mice had greater white blood cell (WBC) counts compared to septic and sham mice in all diet groups. There were no differences in terms of WBC counts in obese septic mice compared to control septic mice at 6 weeks ((2.32 ± 0.283 versus 2.19 ± 0.490) × 10^9^ cell/L), 15 weeks ((1.87 ± 0.275 versus 2.25 ± 0.271 and 1.51 ± 0.212) × 10^9^ cell/L), or at 27 weeks ((2.03 ± 0.235 versus 1.35 ± 0.127 and 1.74 ± 0.330) × 10^9^ cells/L). These results suggest that obesity was not associated with lower WBC counts during infection in our model.

## 4. Discussion

Our study describes murine models of both short-term and prolonged DIO challenged with an infectious stimulus. The highlights of this study are that obesity may reduce incidence of lung inflammation during sepsis and is not associated with changes in hepatic proinflammation and oxidative stress levels. Teoh et al. suggest low adiponectin levels, common in obese individuals, to play a role in greater incidence of sepsis in this population [18]. Based on current literature it is unclear how obesity affects critical illness, with mixed reports from standpoints of clinical and animal studies. Clinical studies suggest that obesity predisposes to increased risk of sepsis [19], with as much as 50% increase in mortality risk [20] and inability to recuperate from critical illness as well as normal weight patients [21, 22]. However, others report obesity to have no effect on mortality rates of critically ill patients and to even be protective, with particularly less incidence of pneumonia [23–25]. For instance, Pickkers et al. reported an inverse relationship between obesity and hospital mortality with obese patients (BMI 30–39.9 kg/m^2^) having the lowest risk of mortality in an observational cohort study of 154,308 ICU patients from Dutch urban and nonurban teaching hospitals [26]. In terms of animal studies, the majority indicate that obesity has negative implications for sepsis outcomes. Strandberg et al. using a* Staphylococcus aureus* (*S. aureus*) model of sepsis reported that mice fed high fat diet had greater incidence of mortality, renal bacterial load, unstable innate immune system (increased serum interleukin-1 receptor antagonist (IL-1Ra), IL-10, and IL-1*β*), and granulocyte dysfunction [11]. Rivera et al. reported that WD feeding increases adhesion of leukocytes in the sinusoids and hepatic venules by 8-fold within the first six hours of sepsis [14]. However, in contrast to our model these mice were fed a fat-inducing diet short term (8 and 3 weeks, resp.). Changes in cytokine production and increase in expression of TNF-*α*, MCP-1 and Intracellular Adhesion Molecule 1 (ICAM-1) have also been reported [14].

The most important finding of our study was that obesity reduced early lung inflammation. Acute lung injury is the leading cause of death in sepsis [27] and requires several different types of therapeutic interventions, contributing to high cost of care associated with the condition [28]. Based on our findings obese septic mice had significantly less airway inflammation therefore protecting these mice from early sepsis-induced lung injury. These results are supported by Wacharasint et al. that conducted a retrospective analysis of body mass and sepsis outcomes. They discovered that obese patients had the lowest 28-day mortality rates and both obese (BMI > 30 kg/m^2^) and overweight (25 < BMI < 30 kg/m^2^) patients had significantly (*P* = 0.03) less lung infection compared to controls (BMI < 25 kg/m^2^) [29]. The mechanism behind these finding is not clear but is thought to be linked to obese patients receiving less norepinephrine, vasopressin, and fluid on day 1 compared to controls [29]. To elucidate the mechanism involved in protection of lung tissues from injury during sepsis, we explored the links between the lung and the upstream hepatic microcirculation.

The second important finding of this study was that reduced lung injury in obese mice was not due to downregulation of hepatic proinflammatory and oxidative stress mediators. Lung and liver functions are very closely linked and hepatic dysfunction directly affects pulmonary function through cytokine production and clearance [30]. However, we found no changes in hepatic proinflammatory cytokine or chemokine levels to account for reduced lung injury. Obesity is usually associated with overload of proinflammatory cytokines including IL-6, TNF-*α*, and MCP-1, MMPs and fatty acid binding protein produced by visceral fat, macrophages, and adipocytes [1]. Obesity is also associated with an increase in oxidative stress and usually a reduction in expression of glutathione [31]. Sastre et al. reported that in mice fed high fat diet, glutathione levels in hepatocytes were 45% lower and oxidized glutathione levels were 54% lower compared to controls [31]. NADPH oxidase catalyzes conversion of oxygen to free radical species that further activates the proinflammatory transcription factor NF-*κ*B. Dandona et al. also reported that in obese individuals a 48-hour fast led to a 50% reduction in the levels of reactive oxygen species and the expression of NADPH oxidase was also reduced [32]. In our model of prolonged high fat diet obesity was not associated with significant differences in proinflammatory cytokine and glutathione levels compared to controls in diet only or septic mice.

Obesity associated damage to the hepatic microcirculation may account for less neutrophil infiltration into lung tissues during sepsis. Based on hematoxylin and eosin stains obese mice had significant steatosis, hepatocyte ballooning, and neutrophil infiltration. Thus, there is a possibility that severe damage to the hepatic microcirculation may trap neutrophils preventing their movement into tissues situated downstream such as the lungs. Further investigation is required to elucidate the mechanism behind the low levels of lung inflammation associated with obesity.

The model described in this study was developed after close examination of the limitations of current animal models of obesity and sepsis. The majority of studies in the literature induce DIO by short exposure to high fat diet (HFD) [11, 14, 33]. Kaplan et al. fed male C57BL/6 mice a HFD for 3 weeks and reported that septic obese mice had lower survival rates and severe lung injury compared to controls [33]. They further reported that lung and liver myeloperoxidase levels were greater in septic obese mice. Similarly Rivera et al. also reported that septic (CLP) mice exposed to 3 weeks of WD had greater hepatic inflammation, TNF-*α*, MCP-1, and ICAM-1 levels [14]. However, for a DIO model to be clinically relevant, long-term exposure to the diet is important. There have been studies that examined implications of prolonged feeding of western diet for sepsis, but the majority utilizes models of endotoxemia [34, 35]. For instance, Pini et al. fed C57BL/6 mice a high fat for 20 weeks and reported an association between obesity and elevated IL-6 levels in response to LPS [34]. As previously mentioned there are several problems with endotoxemia models of sepsis. Firstly, mice are not as sensitive to LPS as humans and therefore require very high dosages to initiate any immune response. Secondly, despite a high dose, they do not demonstrate many aspects of human sepsis and have very different physiological responses and cytokine profiles [36]. Therefore, keeping these limitations in mind we developed a murine model that incorporates important features of both obesity and sepsis present in the clinical settings. We examined both short- and long-term DIO and induced sepsis using a clinically relevant model that closely mirrors the infectious process as seen in humans. The CLP model of sepsis involves surgical induction of a localized infectious focus that disseminates and activates a systemic response in a time-dependent manner. The severity and duration of infection can be regulated making CLP an ideal model of sepsis.

## 5. Conclusion

In summary, in this study we have developed a clinically relevant prolonged model of DIO and described organ specific response to sepsis. We found that prolonged WD feeding induces obesity and reduces glucose tolerance at 15 and 27 weeks. In context of early sepsis, obesity was associated with significantly lower airway inflammation which was not associated with changes in levels of hepatic proinflammatory cytokines or antioxidants. Obesity induced damage to hepatic microvasculature, however, may sequester neutrophils and prevent infiltration into the lungs; however, further research is required to elucidate the specific mechanisms involved.
